# Corrigendum

**DOI:** 10.2471/BLT.26.100126

**Published:** 2026-01-01

In: Guerra Duarte C, de Souza VP, de Oliveira-Sousa G, Gomes Mola MP. Use of snake antivenom in the Region of the Americas: a systematic review. Bull World Health Organ. 2025 Dec 1;104(12):785–798, 

on page 794, the funding section should read as follows:

